# Inferior Vena Cava Thrombus due to Left Inferior Vena Cava and Ulcerative Colitis

**DOI:** 10.1055/s-0038-1673391

**Published:** 2018-10-24

**Authors:** Hirofumi Arai, Akira Mizukami, Kenji Yoshioka, Shunsuke Kuroda, Ryota Iwatsuka, Tatsuya Hayashi, Shigeki Kimura, Akihiko Matsumura

**Affiliations:** 1 Department of Cardiology, Kameda Medical Center, Kamogawa City, Chiba, Japan

**Keywords:** venous thrombosis, thrombosis, hypercoagulability

## Abstract

A 29-year-old man with diarrhea and abdominal pain for 2 weeks presented with new-onset left back pain. Contrast-enhanced computed tomography (CT) showed a left inferior vena cava (IVC) crossing over the aorta, and thrombus in the IVC and left renal vein. Colonoscopy and biopsy for assessment of diarrhea and abdominal pain provided a diagnosis of ulcerative colitis. Stasis of blood flow due to left IVC crossing over the aorta, and hypercoagulability due to ulcerative colitis influenced thrombus formation.

A 29-year-old man with diarrhea and abdominal pain for 2 weeks presented to the emergency department with new-onset left back pain. He had no significant past medical history. Contrast-enhanced computed tomography (CT) showed a left inferior vena cava (IVC) that crossed over the abdominal aorta, with giant thrombus in the IVC and left renal vein distal to the crossover point ( Figs. 1 , 2 ). There were no signs of deep vein thrombosis or malignancy. He had no abnormal findings on autoantibody, protein C/S, and antithrombin III testing. We started anticoagulation with apixaban. Colonoscopy and biopsy were performed for assessment of diarrhea and abdominal pain, resulting in a diagnosis of ulcerative colitis, and mesalazine was started. Thrombus disappeared on contrast-enhanced CT at 1-month follow-up. We continued anticoagulation thereafter. It was thought that stasis of blood flow due to left IVC crossing over the aorta and hypercoagulability due to ulcerative colitis influenced thrombus formation. There are no reports to date of IVC thrombus due to left IVC and ulcerative colitis. Left IVC is a rare anomaly, and can be a cause of thrombus formation.

**Fig. 1:**
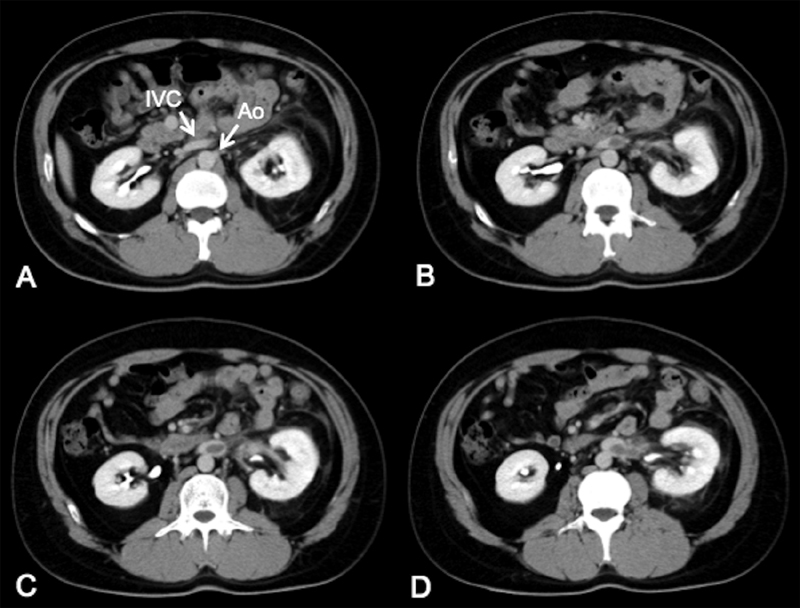
( **A** – **D** ) Axial view of contrast-enhanced computed tomography from cranial to caudal. Left inferior vena cava (IVC) crosses over the abdominal aorta, and IVC thrombus is shown.

**Fig. 2:**
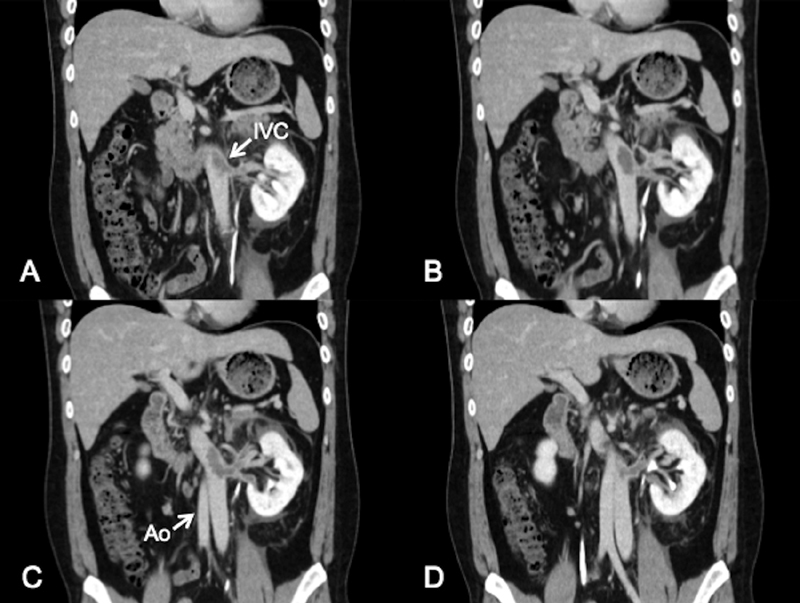
( **A** – **D** ) Coronal view of contrast-enhanced computed tomography from abdominal to dorsal. Giant thrombus appears in left inferior vena cava and left renal vein distal to the crossover point.

